# Polarization-Resolved Digital Holographic Sensing for Simultaneous Morphological and Polarimetric Characterization of Microparticles

**DOI:** 10.3390/s26196097

**Published:** 2026-09-25

**Authors:** Yang Chen, Xiang Xu, Ruishan Xiao, Yufan Xuan, Xiaojing Shi, Jingjing Liu, Jun Wang

**Affiliations:** School of Optoelectronic Science and Intelligent Instrumentation, Xi’an University of Technology, Xi’an 710048, China; cyang622@163.com (Y.C.); xiaoruishan0421@163.com (R.X.); xuanyf086@163.com (Y.X.); 2240220113@stu.xaut.edu.cn (X.S.); jingjingliu@xaut.edu.cn (J.L.); wangjun790102@xaut.edu.cn (J.W.)

**Keywords:** optical sensing, digital holography, polarization imaging, microparticle characterization, degree of linear polarization, refractive index

## Abstract

**Highlights:**

**What are the main findings?**

- The PRDH sensing system co-registers three-dimensional position, morphological descriptors, and linear-polarization parameters for individual microparticles within the same particle field.
- The measured group-mean DoLP differs among the investigated particle groups; numerical calculations illustrate the angle-dependent effects of particle size and refractive index.

**What are the implications of the main findings?**

- The method provides an integrated optical-sensing framework for particle-resolved multiparameter characterization in three-dimensional particle fields.
- Linear-polarization measurements complement projected morphology, while their interpretation requires consideration of size, shape, orientation, and measurement geometry.

**Abstract:**

Multiparameter optical sensing of micrometer-sized particles requires morphological and optical responses to be resolved for the same particles. We present a polarization-resolved digital holographic (PRDH) system combining digital in-line holography with division-of-focal-plane polarimetry. Numerical refocusing associates the three-dimensional location of each resolved particle with its equivalent diameter, major and minor axes, perimeter, projected area, and shape factor, together with the linear Stokes parameters *S*_0_, *S*_1_, and *S*_2_ and the degree of linear polarization (DoLP). For three standard glass-microsphere groups, the average absolute relative deviation between group-mean measured diameters and the nominal reference values was approximately 5.5%. The 5 and 10 micrometer borosilicate groups had mean DoLP values of 0.849 and 0.843, respectively, while other sample groups exhibited different responses. An exploratory regression against the adopted nominal refractive-index values is reported as a conditional group-level description, not as a refractive-index calibration. T-matrix calculations at 520 nm illustrate angular variations with size and refractive index without quantitatively reproducing the complete holographic measurement. Ice-cloud-chamber measurements further demonstrate the application of the particle-resolved analysis. PRDH thus provides associated positional, projected morphological, and linear-polarization information within a three-dimensional particle field.

## 1. Introduction

Micrometer-sized particles are ubiquitous in atmospheric environments, biopharmaceutical formulations, and industrial processes. Their size, morphology, spatial distribution, and optical properties are often required simultaneously because these quantities describe complementary aspects of particle state and composition. In atmospheric research, particle concentration, size distribution, and morphology are important for pollution monitoring, exposure assessment, and cloud microphysical studies [1,2,3]. In biopharmaceutical applications, the number, size, morphology, and optical characteristics of protein aggregates, silicone-oil droplets, and glass fragments are relevant to product quality and stability [4]. These demands are driving particle sensing from single-parameter measurements toward multiparameter characterization at the individual-particle level [5,6].

Existing particle-measurement techniques provide different subsets of this information. Optical particle counters and single-particle light-scattering instruments rapidly estimate particle size and number concentration [7,8], while optical array probes, microscopic imaging, and micro-flow imaging provide two-dimensional size and shape information [3,9,10]. Electron microscopy, Raman spectroscopy, and Fourier-transform infrared spectroscopy can provide finer morphological or material-composition information but generally require different measurement configurations and do not inherently provide three-dimensional particle localization [4,6]. Polarimetric measurements offer a complementary optical dimension because the polarization response of scattered light depends on refractive index, shape, internal structure, orientation, and scattering geometry [11,12]. Ensemble polarimetry and lidar have demonstrated the value of polarization for characterizing suspended particles and aerosols [13,14,15], whereas single-particle polarimetry can retain particle-to-particle heterogeneity. For example, microfluidic or flow-based systems have been used to guide particles through a defined polarization-scattering region for individual-particle analysis [16,17]. However, these approaches typically rely on constrained flow channels, fixed scattering volumes, or limited focal depths.

Digital holography records a particle field in a hologram that can be numerically propagated to refocus particles at different depths [18,19,20]. Polarization-sensitive holography has long provided additional optical information [21,22,23,24,25,26,27]. In particle measurements, Öhman et al. demonstrated polarization-resolved dual-view holography for three-dimensional localization and tracking [28], followed by nanoparticle identification and sizing combined with T-matrix modeling [29]. Mueller–Gabor holographic microscopy extends in-line reconstruction to volumetric Mueller-matrix information [30]. More recently, a submersible single-shot lensless polarimetric holographic system has been used for microplastic classification in field-collected stream water [31], and polarization digital holographic microscopy has supported automated multiparameter microplastic characterization [32]. These studies establish that single-shot acquisition and joint morphology–polarization analysis are not, by themselves, new capabilities.

Particle-hologram analysis also includes computer-vision methods for autofocus, segmentation, and contour measurement, as well as learning-based reconstruction and classification. Dense-U-Net addresses reconstruction of three-dimensional particle fields [33], whereas HoloForkNet reconstructs multiple object planes through a multibranch network [34]. Learning-based polarimetric holography has been developed for microplastic classification [35], and metasurface vector holography with neural-network decoding enables single-shot full-Stokes detection [36]. Reviews describe the broader roles of deep learning in phase recovery and digital holography [37,38]. These methods offer complementary routes to reconstruction, denoising, and recognition; supervised approaches require training data appropriate to the task and measurement conditions.

The present work focuses on a single-view in-line measurement arm with a 5× telecentric lens and simultaneous four-orientation division-of-focal-plane (DoFP) detection, supplemented by an incident-polarization monitoring arm. A deterministic reconstruction and analysis pipeline associates each resolved particle with its three-dimensional location, two-dimensional projected morphology, and linear-polarization descriptors. Relative to dual-view systems, this layout avoids inter-view registration but provides fewer angular constraints; relative to Mueller-matrix imaging, it obtains less complete polarization information in exchange for simultaneous linear-polarization acquisition. Its role is quantitative particle characterization without task-specific supervised training, rather than a new principle of polarization holography. Standard microsphere sizing, calibrated linear-polarization measurements, illustrative scattering calculations, and an ice-cloud-chamber application define the scope of the evaluation.

## 2. Measurement Principles

### 2.1. Polarization Measurement and Refractive-Index Sensitivity

For an interface between two dielectric media, the Fresnel coefficients for s- and p-polarized light depend on the refractive indices and incidence geometry [39]:

(1) $$r_{s}=\frac{n_{1}\cos\theta_{i}-n_{2}\cos\theta_{t}}{n_{1}\cos\theta_{i}+n_{2}\cos\theta_{t}}$$

(2) $$r_{p}=\frac{n_{2}\cos\theta_{i}-n_{1}\cos\theta_{t}}{n_{2}\cos\theta_{i}+n_{1}\cos\theta_{t}}$$

where *n*_1_ and *n*_2_ are the refractive indices of the media, *θ_i_* is the angle of incidence, and *θ_t_* is the angle of refraction, satisfying Snell’s law:

(3) $$n_{1}\sin\theta_{i}=n_{2}\sin\theta_{t}$$

(4) $$\cos\theta_{t}=\sqrt{1-{\left(\frac{n_{1}}{n_{2}}\right)}^{2}{\left(\sin\theta_{i}\right)}^{2}}$$

The Fresnel relations illustrate, at the interface level, why refractive-index contrast can modify the relative amplitudes of orthogonal polarization components. For an incident field resolved into p- and s-polarized components, the reflected components can be written as

(5) $$E_{ref}=\left[\begin{array}{c} E_{p}^{ref}\\ E_{s}^{ref} \end{array}\right]=\left[\begin{array}{c} r_{p}E_{p}^{in}\\ r_{s}E_{s}^{in} \end{array}\right]$$

Here, *E*_p_ and *E*_s_ denote the p- and s-polarized electric-field components, and *r*_p_ and *r*_s_ are the corresponding Fresnel reflection coefficients. These relations are introduced only to illustrate the refractive-index sensitivity of polarization at an optical interface; the polarization-resolved digital holographic (PRDH) measurements in this study are not interpreted as a direct Fresnel-reflection inversion.

The polarization state of a partially polarized optical field can be represented by the four Stokes parameters:

(6) $$\begin{array}{c} S_{0}=\langle {\left|E_{p}\right|}^{2}\rangle +\langle {\left|E_{s}\right|}^{2}\rangle =A_{p}^{2}+A_{s}^{2}\\ S_{1}=\langle {\left|E_{p}\right|}^{2}\rangle -\langle {\left|E_{s}\right|}^{2}\rangle =A_{p}^{2}-A_{s}^{2}\\ S_{2}=2Re\langle E_{p}E_{s}^{*}\rangle =2A_{p}A_{s}\cos\left(\phi_{p}-\phi_{s}\right)\\ S_{3}=2Im\langle E_{p}E_{s}^{*}\rangle =2A_{p}A_{s}\sin\left(\phi_{p}-\phi_{s}\right) \end{array}$$

Here, *E*_p_ and *E*_s_ are orthogonal electric-field components, *A*_p_ and *A*_s_ are their amplitudes, and *ϕ*_p_ and *ϕ*_s_ are their phases; the asterisk denotes complex conjugation. Re and Im denote the real and imaginary parts. For a partially polarized field, the field products are understood as time or ensemble averages; the amplitude-phase expressions describe the corresponding coherent-field case.

For intensity-based polarization detection, the Stokes parameters can be expressed in terms of analyzer-resolved intensities [40]:

(7) $$S=\left(\begin{array}{c} S_{0}\\ S_{1}\\ S_{2}\\ S_{3} \end{array}\right)=\left(\begin{array}{c} I_{0^{{}^{\circ}}}+I_{90^{{}^{\circ}}}\\ I_{0^{{}^{\circ}}}-I_{90^{{}^{\circ}}}\\ I_{45^{{}^{\circ}}}-I_{135^{{}^{\circ}}}\\ I_{R}-I_{L} \end{array}\right)$$

In Equation (7), *I*_0°_, *I*_45°_, *I*_90°_, and *I*_135°_ denote intensities measured through linear analyzers at the indicated orientations, while *I*_R_ and *I*_L_ denote right- and left-circular analyzer intensities under the stated *S*_3_ sign convention. The present camera records only the four linear analyzer states; the circular states are included to define the complete Stokes vector and are not measured here.

Based on the complete Stokes vector, the degree of polarization (DoP) is defined as

(8) $$DoP=\frac{\sqrt{S_{1}^{2}+S_{2}^{2}+S_{3}^{2}}}{S_{0}}$$

When only the linear polarization components are considered, the degree of linear polarization (DoLP) is calculated as follows [41]:

(9) $$DoLP=\frac{\sqrt{S_{1}^{2}+S_{2}^{2}}}{S_{0}}$$

DoLP ranges from 0 to 1 for a physically admissible Stokes vector and is calculated from *S*_0_, *S*_1_, and *S*_2_ using Equation (9). Its evaluation does not require *S*_3_, but it cannot determine the total DoP or distinguish a circularly polarized contribution from an unpolarized contribution. Linear incident polarization does not ensure that the detected *S*_3_ is zero. Because the hologram includes both the transmitted reference field and the particle-scattered field, the retrieved DoLP also depends on illumination, collection geometry, and reconstruction. A reduced DoLP is therefore not, by itself, a measurement of particle depolarization or a unique indicator of refractive index.

The Fresnel relations provide interface-level motivation only. Particle scattering is described by electromagnetic scattering theory; Section 5 uses a simplified T-matrix calculation to examine changes in angular polarization response with particle parameters.

### 2.2. Digital In-Line Holographic Reconstruction

Digital in-line holography (DIH) provides a compact route to volumetric particle sensing because a single recorded hologram can be numerically propagated to multiple axial planes. When a plane wave illuminates a particle, the particle-scattered field interferes with the unscattered transmitted field, and the resulting interference pattern is recorded as a hologram. The object field can then be numerically reconstructed using scalar diffraction propagation. For a measurement volume containing multiple particles, the reconstructed field at an axial distance *Z*_r_ can be written as

(10) $$U_{R}\left(u,v\right)=\frac{1}{i\lambda }\iint_{\infty }R\left(x,y\right)I_{H}\left(x,y\right)\frac{exp\left(ik\sqrt{{\left(u-x\right)}^{2}+{\left(v-y\right)}^{2}+Z_{r}^{2}}\right)}{\sqrt{{\left(u-x\right)}^{2}+{\left(v-y\right)}^{2}+Z_{r}^{2}}}dxdy$$

Here, *λ* is the wavelength, i is the imaginary unit, *k=2π/λ* is the wavenumber, *R(x,y)* is the reference field, *I*_H_*(x,y)* is the recorded hologram intensity, *(x,y)* and *(u,v)* are transverse coordinates in the recording and reconstruction planes, and *Z*_r_ is the numerical propagation distance. Propagation through successive planes brings different particles into focus [42]. The reconstruction coordinate must be distinguished from an independently calibrated object-space displacement in an imaging system.

A minimum-area bounding rectangle is fitted to each segmented contour using a rotating-calipers procedure [43,44]. Its long and short sides define the reported major and minor axes. In Equation (11), *A* is the projected area, *L* is the major-axis length, and *P* is the contour perimeter; the area-equivalent diameter is $d_{eq}=\sqrt{4A/\pi }$. The dimensionless shape descriptor is defined as [45,46]

(11) $$F=\frac{L\times P}{A}$$

An ideal continuous circular projection gives *F* = 4. Values from pixelated, segmented images depend on contour estimation and are not an independent measure of three-dimensional sphericity.

## 3. Materials and Methods

### 3.1. PRDH Sensing System

The PRDH sensing system integrates digital in-line holography with division-of-focal-plane polarimetry so that holographic and linear-polarization information is recorded under the same illumination condition. This architecture is designed to support particle-resolved retrieval of morphology and polarization within a three-dimensional measurement volume. The optical layout is shown in Figure 1a.

A 520 nm pulsed laser is expanded by L1 and collimated by L2 to provide approximately uniform illumination. The beam then passes through the linear polarizer L3 (extinction ratio > 1000:1). Beam splitter L4 divides the polarized beam into a monitoring arm and a measurement arm. Both DoFP1 and DoFP2 were MER2-503-36U3M POL polarization cameras (DAHENG IMAGING, Beijing, China). The monitoring arm is recorded by DoFP1 to monitor the incident polarization state, while the measurement arm illuminates the particle field. The particle-scattered field and the unscattered transmitted reference field are collected by a 5× telecentric lens (WWK50-102-111V3, COOLENS, Shenzhen, China; 102 mm working distance; distortion 0.16%; telecentricity 0.18°) and recorded by DoFP2. DoFP2 incorporates a Sony IMX264MZR global-shutter CMOS polarization image sensor (Sony Semiconductor Solutions Corporation, Atsugi, Kanagawa, Japan) with a 5-megapixel resolution and simultaneously records analyzer-resolved intensities at 0°, 45°, 90°, and 135° at up to 36 frames/s at full resolution. The simultaneous four-orientation acquisition provides the intensity information required for linear Stokes and DoLP retrieval, while the holographic interference pattern provides the information required for numerical refocusing and three-dimensional localization [47].

Images were stored as uncompressed monochrome bitmap (BMP) files with an 8-bit saved intensity scale. The exposure time was 100 μs. The laser wavelength was 520 nm, with reported peak and average powers of 200 and 110 mW, respectively; acquisition used an external hardware trigger. The saved-image bit depth should not be confused with the internal sensor analog-to-digital converter (ADC) specification.

### 3.2. DoFP Calibration and Verification

Before particle measurements, the four analyzer channels were calibrated for dark offsets, spatial response nonuniformity, and relative polarimetric response. The measured intensity vector is modeled as

(12) i=MS+ε

where

(13) $$i={\left[\begin{array}{cccc} I_{0} & I_{45} & I_{90} & I_{135} \end{array}\right]}^{T},\;S={\left[\begin{array}{ccc} S_{0} & S_{1} & S_{2} \end{array}\right]}^{T}$$

**M** is the 4 × 3 instrument matrix, and ***ε*** is the measurement-noise vector. The calibration concerns the three linear Stokes components, not full-Stokes recovery.

Dark frames acquired at the experimental exposure and gain were averaged pixel by pixel to obtain *D_k_(x,y)*. Under uniform illumination, a high-extinction-ratio linear polarizer was rotated through orientations spanning 0–180°, and responses were averaged to obtain flat fields *F_k_(x,y)*. Each flat field was normalized by its spatial median to correct spatial nonuniformity; residual relative channel gains were included in the subsequent instrument-matrix calibration. The corrected intensities were calculated as

(14) $$I_{k}^{cal}\left(x,y\right)=\frac{I_{k}^{raw}\left(x,y\right)-D_{k}\left(x,y\right)}{F_{k}\left(x,y\right)+\varepsilon_{reg}}$$

where *k* denotes the analyzer orientation, and *ε*_reg_ is a small positive scalar denominator regularizer, distinct from the noise vector in Equation (12).

Known linear input orientations *ϕ_j_* were then used to obtain mean channel responses in a uniformly illuminated region. The normalized reference Stokes vectors were written as

(15) $$S^{\left(j\right)}=\left[\begin{array}{c} 1\\ \cos{2\phi }_{j}\\ \sin{2\phi }_{j} \end{array}\right]$$

This normalized representation assumes stable illumination during the angular scan; a common intensity scale is absorbed into **M**. With reference states and corresponding corrected responses assembled as columns of **S** and **Y**, respectively, the least-squares estimate is

(16) $$M=YS^{T}{\left(SS^{T}\right)}^{-1}$$

For a full-column-rank instrument matrix, the corresponding least-squares inverse is

(17) $$P={\left(M^{T}M\right)}^{-1}M^{T}$$

The calibrated linear Stokes components were recovered as

(18) $$\hat{S}\left(x,y\right)=Pi^{cal}\left(x,y\right)$$

DoLP was then calculated using Equation (9), and the same instrument-matrix procedure was applied to the particle measurements.

Figure 2 compares the DoLP of the linearly polarized illumination in a particle-free region before and after correction. The displayed mean changes from 0.969 to 1.001, approaching the expected value of unity. The slight estimate above unity reflects measurement and estimation error, not a physically super-unity DoLP. This check supports the correction of channel-response mismatch near a fully linearly polarized state; it does not independently validate the entire DoLP range or every particle-reconstruction error. Holographic reconstruction, image processing, and parameter extraction were performed using custom-written programs in MATLAB R2024b (The MathWorks, Inc., Natick, MA, USA).

### 3.3. Particle Samples and Evaluation Design

Droplets, borosilicate glass microspheres, soda-lime glass microspheres, and irregular sand grains were investigated. The glass size standards were Thermo Scientific 9000-series particles (Thermo Fisher Scientific, Fremont, CA, USA). The values 5.1 ± 0.5, 10.0 ± 1.0, and 49.0 ± 1.4 μm were taken from the supplied size specifications; the ± terms are not standard deviations calculated from our measurements. The groups are abbreviated as 5, 10, and 50 μm. Manufacturer-supplied size specifications and experimentally measured group means serve different purposes and are reported separately.

The 5 and 10 μm borosilicate groups share a material designation, whereas the cross-material groups differ in size, shape, and potentially surface properties. Refractive indices of 1.33, 1.47, 1.50, and 1.52 were used as the nominal values for the exploratory comparison, not as independently measured optical constants at 520 nm. A material designation alone does not establish a batch-specific index, and a single value for heterogeneous sand is an approximation. Regression against these values is conditional on their assignment and cannot establish an intrinsic material calibration.

Particles were introduced using a lateral airflow and recorded while moving through the measurement field. Dilute loading reduced the likelihood of overlapping images, and the analysis concerned particles that could be individually resolved after reconstruction. All groups used the same optical system and processing framework. Airflow does not eliminate gravitational settling or impose a known orientation on irregular particles, and refocusing does not guarantee separation of all overlapping holograms. These effects remain sources of variability, particularly for sand grains.

For each recording sequence, the final ten background frames were averaged to form a reference for hologram preprocessing. The particle mask obtained after reconstruction and segmentation restricted subsequent statistics to the selected region. Background subtraction reduces stationary contributions, whereas masking excludes surrounding background pixels; neither removes all coherent interference or reconstruction artifacts.

## 4. Results

### 4.1. Morphological Parameter Retrieval and Particle-Size Validation

Each hologram was numerically propagated to 35 reconstruction planes spanning approximately 5 mm along the optical axis, around a propagation distance of 170 mm. This sampling interval defines the numerical search grid; it is not an independently measured axial localization accuracy.

The transfer function employed by the convolution method is

(19) $$H\left(z\right)=\exp\left(i2\pi z\sqrt{\left(\frac{1}{\lambda^{2}}-{\left(\frac{x}{X\delta }\right)}^{2}-{\left(\frac{y}{Y\delta }\right)}^{2}\right)}\right)$$

Here, *H* is the propagation transfer function, i is the imaginary unit, *λ* is the wavelength, *z* is the propagation distance, *δ* is the sampling pitch, and *X* and *Y* are the horizontal and vertical array sizes. In Equation (19), *x* and *y* denote centered frequency-bin indices, so *x/(Xδ)* and *y/(Yδ)* are spatial frequencies. These discrete indices differ from the spatial coordinates used in Equation (10). All lengths used in propagation are expressed in consistent units.

A focus metric was evaluated along the reconstruction stack to select a focal plane for each resolvable particle. The selected plane provides its axial coordinate, while segmentation in that plane provides the projected morphology. The discrete reconstruction spacing and the width of the focus response limit localization; no independent axial-position accuracy is inferred from the reconstruction step alone.

Morphological parameters were then extracted from the in-focus particle image. Figure 3 shows representative processing results for (a) a 10 μm borosilicate microsphere, (b) a 50 μm soda-lime glass microsphere, and (c) a sand grain. The reconstructed in-focus images are shown in Figure 3(a-3,b-3,c-3).

Each in-focus particle was segmented by intensity thresholding followed by morphological opening and closing to suppress isolated pixels and smooth the particle boundary. When enclosed cavities remained in the segmented region, hole filling was applied to obtain a continuous particle mask. The resulting masks are shown in Figure 3(a-4,b-4,c-4).

Table 1 lists the three-dimensional coordinates and projected morphological descriptors of the three representative particles in Figure 3. The descriptors include equivalent-area diameter, minor and major axes, perimeter, area, and shape factor. These measurements describe two-dimensional projections at the selected focus, rather than a complete reconstruction of each particle surface.

For the three nominal-size microsphere groups, the measured mean diameters were 4.565, 10.304, and 50.463 μm, respectively. Relative to the supplied central sizes of 5.1, 10.0, and 49.0 μm, the absolute relative deviations of the group means were 10.49%, 3.04%, and 2.99%. Their arithmetic mean was approximately 5.5%. This metric is an average of three group-mean deviations; it is not a mean absolute error over individual particles, an uncertainty interval, or a guarantee of the same accuracy for irregular particles. The corresponding particle-size measurements and distributions are shown in Figure 4.

For context, Di et al. reported a measured diameter of 10.32 μm for a nominal 10 μm particle [48], corresponding to a 3.2% deviation. Walcutt et al. reported a mean diameter error below 2% for 50 μm standard beads and errors of approximately 3–10% for more complex particle assemblages [49]. The present 10 and 50 μm groups have deviations of 3.04% and 2.99%, respectively. Differences in samples, reference values, optics, and analysis prevent these literature values from constituting a controlled head-to-head benchmark.

Using the 1.5 × interquartile range (IQR) rule, the lower and upper fences were 2.959–6.103, 7.440–13.198, and 45.356–55.849 μm for the three groups, respectively; no measured values lay outside these fences. Here, IQR = *Q*_3_ − *Q*_1_, and the fences are *Q*_1_ − 1.5 × IQR and *Q*_3_ + 1.5 × IQR. The absence of outliers under this rule does not rule out systematic error. Box-plot whiskers describe the observed values within the fences. Required sizing accuracy depends on the application and must be assessed separately from polarization-based interpretation.

The mean shape factors for the spherical and irregular particle groups were 3.67 and 8.82, respectively (Figure 5). For the definition given in Equation (11), an ideal continuous circular projection gives *F* = 4. Pixel sampling, segmentation, and perimeter estimation can shift measured values, particularly for small particles. The measured separation therefore supports a distinction in projected morphology within these data, but the value 3.67 is not by itself a calibrated measure of shape accuracy.

The standard-particle measurements provide an assessment of group-mean sizing and projected shape discrimination. The following analysis considers linear-polarization information associated with the reconstructed particles; these validation tasks are distinct.

### 4.2. Polarimetric Characterization

The four analyzer channels were processed with the calibrated instrument matrix to obtain *S*_0_, *S*_1_, and *S*_2_, and DoLP was calculated according to Equation (9) for the selected particle regions. Numerical refocusing and segmentation were used to associate the polarization measurements with each resolvable particle. Equation (7) describes ideal analyzers, whereas the instrument-matrix inversion accounts for the measured channel responses. The results characterize the detected particle-associated optical field, which may include coherent background and reconstruction effects, rather than an isolated scattering field measured over all angles.

The mean DoLP values of droplets, 5 μm borosilicate microspheres, 10 μm borosilicate microspheres, sand grains, and soda-lime microspheres were 0.938, 0.849, 0.843, 0.797, and 0.763, respectively. Representative DoLP maps of these particle groups are shown in Figure 6. The difference between the two borosilicate groups was 0.006. This close agreement under the present conditions does not establish size independence. Cross-material comparisons also involve differences in size, shape, orientation, and surface properties, so the group differences cannot be attributed exclusively to refractive index.

An exploratory least-squares regression was performed using the five group-mean DoLP values and the adopted nominal refractive indices (Figure 7f):

(20) $$DoLP=-0.852\;n+2.080,R^{2}=0.926$$

The slope was −0.852 (95% confidence interval: −1.293 to −0.411), and the intercept was 2.080 (95% confidence interval: 1.436 to 2.724), with *R*^2^ = 0.926. The residuals ranged from −0.02218 to 0.02122 and occurred on both sides of zero. These intervals are conditional on the assigned indices and the ordinary least-squares assumptions; they do not include refractive-index uncertainty or account for cross-group physical confounding. The fit contains five group means, only four distinct nominal index levels, and three residual degrees of freedom. It is a descriptive association under the present measurement conditions, not a universal DoLP–index calibration or a demonstration of causal refractive-index dependence.

### 4.3. Application Demonstration in an Ice-Cloud-Chamber Particle Field

Measurements in an ice-cloud chamber extend the application demonstration beyond standard microspheres. The same PRDH acquisition and processing framework was used to associate refocused particle images with linear-polarization information. The droplet and ice-crystal groups refer to the group assignments in this chamber dataset; these measurements do not establish an independently validated phase classifier.

The mean DoLP values for the chamber droplet and ice-crystal groups were 0.857 and 0.742, respectively, giving a difference of 0.115 (Figure 8). These means describe the two groups under the chamber measurement conditions. The difference can reflect several coupled factors, including morphology, size, orientation, optical properties, and collection geometry; it is not assigned solely to refractive index.

The chamber results illustrate application to a more heterogeneous particle field. They are not an additional controlled test of the nominal-index regression, a cross-method benchmark, or evidence that DoLP alone identifies particle phase.

## 5. T-Matrix Simulation and Discussion

T-matrix calculations were used as an illustrative parameter study of polarization scattering by model spheres [50]. The framework relates incident and scattered multipole coefficients and permits one parameter to be varied while the others are held fixed. It provides context for interpreting polarization sensitivity but is not a direct forward model of the complete PRDH measurement.

The incident and scattered fields are expressed as

(21) $$E^{inc}\left(R\right)=\sum_{n=1}^{n_{max}}\sum_{m=-n}^{n}\left[a_{mn}RgM_{mn}\left(kR\right)+b_{mn}RgN_{mn}\left(kR\right)\right]$$

(22) $$E^{sca}\left(R\right)=\sum_{n=1}^{n_{max}}\sum_{m=-n}^{n}\left[p_{mn}M_{mn}\left(kR\right)+q_{mn}N_{mn}\left(kR\right)\right],\left|R\right|>r_{0}$$

Here, **E**^inc^ and **E**^sca^ denote the incident and scattered electric fields; **R** is the position vector; *k* is the wavenumber in the surrounding medium; and *r*_0_ is the radius of a sphere enclosing the particle. Rg**M***_mn_* and Rg**N***_mn_* denote regular vector spherical wave functions, whereas **M***_mn_* and **N***_mn_* denote outgoing functions. The indices *n* and *m* denote the multipole order and azimuthal index, respectively; *n*_max_ is the truncation order, and primed indices label summed modes. Coefficients *a_mn_* and *b_mn_* describe the incident field, and *p_mn_* and *q_mn_* describe the scattered field.

(23) $$p_{mn}=\sum_{n^{\prime }=1}^{n_{max}}\sum_{m^{\prime }=-n^{\prime }}^{n^{\prime }}\left[T_{mnm^{\prime }n^{\prime }}^{11}a_{m^{\prime }n^{\prime }}+T_{mnm^{\prime }n^{\prime }}^{12}b_{m^{\prime }n^{\prime }}\right]$$

(24) $$q_{mn}=\sum_{n^{\prime }=1}^{n_{max}}\sum_{m^{\prime }=-n^{\prime }}^{n^{\prime }}\left[T_{mnm^{\prime }n^{\prime }}^{21}a_{m^{\prime }n^{\prime }}+T_{mnm^{\prime }n^{\prime }}^{22}b_{m^{\prime }n^{\prime }}\right]$$

The scattered expansion coefficients depend linearly on the incident coefficients through the T-matrix, as expressed in Equations (23)–(25).

(25) $$\left[\begin{array}{c} p\\ q \end{array}\right]=T\left[\begin{array}{c} a\\ b \end{array}\right]=\left[\begin{array}{cc} T^{11} & T^{12}\\ T^{21} & T^{22} \end{array}\right]\left[\begin{array}{c} a\\ b \end{array}\right]$$

The T-matrix is then written as follows:

(26) $$T=-RgQ{\left[Q\right]}^{-1}$$

The blocks **T**^11^, **T**^12^, **T**^21^, and **T**^22^ connect the two sets of incident and scattered multipole coefficients. In Equation (26), **Q** and Rg**Q** are boundary-condition matrices in the extended boundary-condition formulation, where Rg denotes the regular-wave form. These matrices depend on particle geometry and optical constants. The notation is distinct from the Stokes parameters and the camera response matrix.

The scattered Stokes vector is related to the incident Stokes vector through the 4 × 4 scattering Mueller matrix in Equation (27). This matrix describes the modeled scatterer and must be distinguished from the 4 × 3 instrument-response matrix used to calibrate the DoFP camera.

(27) $$S^{\prime }=MS$$

For the spherical-particle model, the Mueller matrix has the symmetry shown in Equation (28), with the polarization reference axes defined relative to the scattering plane. This symmetry is a property of the assumed scattering model and is not a general description of arbitrarily oriented irregular particles.

(28) $$M\left(\theta \right)=\left[\begin{array}{cccc} F_{11} & F_{12} & 0 & 0\\ F_{12} & F_{22} & 0 & 0\\ 0 & 0 & F_{33} & F_{34}\\ 0 & 0 & {-F}_{34} & F_{44} \end{array}\right]$$

The calculations use a wavelength of 520 nm. For the size comparison, the adopted real refractive index is fixed at 1.47, and the nominal diameters are 5, 10, and 15 μm. The first two sizes correspond to the nominal experimental borosilicate groups; 15 μm is an additional model condition. For the index comparison, the nominal diameter is fixed at 5 μm, and the real indices are 1.33, 1.47, 1.50, and 1.52. The imaginary index is set to zero. A Hansen–Travis size distribution with an effective variance of 0.10 and seven Gaussian-quadrature nodes is used consistently for size averaging. The distribution width and quadrature count are calculation settings, not a fit to measured size distributions or an independently demonstrated convergence criterion. The adopted indices are nominal model inputs rather than verified batch-specific values at 520 nm.

Figure 9 shows that both size and refractive index alter the angular DoLP response. Their relative influence depends on the scattering angle and the parameter interval; curve crossings preclude a universal ordering or a claim that DoLP is generally size-independent. These calculations do not reproduce the finite angular collection, coherent reference interference, imaging, and reconstruction of the experimental system. They therefore illustrate parameter sensitivity rather than quantitatively validating the measured group means or the fitted slope.

For complex particles, three-dimensional shape, orientation, surface structure, absorption, and the collection geometry can additionally change the polarization response. The spherical, nonabsorbing model does not quantitatively predict the behavior of sand grains or ice particles. The present measurements should consequently be interpreted as particle-associated morphology and linear-polarization measurements, with material interpretation limited by these coupled effects.

The implemented analysis requires approximately 25 s to process one complete hologram through reconstruction and parameter extraction. In contrast, the experimental exposure time is 100 μs, and the four DoFP analyzer channels are acquired simultaneously in a single exposure. Therefore, the present limitation is mainly associated with the computational processing stage rather than image acquisition: holographic reconstruction and particle analysis are currently performed offline rather than in real time. Parallel propagation and reduced reconstruction searches may improve computational efficiency. Beyond computational acceleration, future development of the PRDH system may also include automated reconstruction and deep learning-based reconstruction. Automated processing could further integrate particle detection, focusing, segmentation, and parameter extraction, while deep learning methods could be explored to estimate particle focal positions or directly map recorded holograms to refocused particle images, thereby reducing repeated multi-plane numerical propagation. These directions will be investigated in future work.

A DoFP 2 × 2 analyzer mosaic assigns one native pixel to each of the four analyzer orientations. Each directly sampled analyzer image therefore has half the native sensor sampling count along each axis and one quarter of its pixel count. For the stated optical magnification, the corresponding object-space analyzer-channel pitch is approximately 1.38 μm. This sampling pitch is not an independently measured optical resolution, and interpolation cannot restore spatial information that was not sampled.

Coherent background, shot and read noise, channel mismatch, twin-image contributions, and segmentation uncertainty can affect the retrieved quantities. A background-subtracted hologram can contain signed values and should not itself be interpreted as a nonnegative optical intensity in a Stokes formula. Likewise, DoLP values should not be background-subtracted as if they were linear intensities. The present background treatment and calibration reduce selected contributions but do not provide a complete uncertainty budget.

The four linear analyzers provide no sensitivity to *S*_3_. Consequently, DoLP does not specify the full polarization state, and a decrease in DoLP cannot distinguish circular polarization from unpolarized content. Linear incident light does not guarantee that *S*_3_ remains zero after interaction with a particle. Full-Stokes snapshot systems are possible [36], but would require additional polarization-analysis capability beyond the present instrument.

## 6. Conclusions

A single-view PRDH framework was implemented to associate three-dimensional particle positions and projected morphological descriptors with *S*_0_, *S*_1_, *S*_2_, and DoLP. Simultaneous DoFP acquisition avoids sequential analyzer exposures, while numerical refocusing supports particle-resolved analysis within a three-dimensional field. Standard particle group means gave an average absolute relative diameter deviation of approximately 5.5%. The 5 and 10 μm borosilicate groups had mean DoLP values of 0.849 and 0.843. Cross-material DoLP differences and the conditional nominal-index regression provide a descriptive optical comparison, subject to confounding and material-parameter uncertainty. T-matrix calculations illustrate size- and index-dependent angular responses without constituting a quantitative instrument calibration. Chamber measurements further demonstrate application to droplet and ice-crystal groups, with mean DoLP values of 0.857 and 0.742. These results support multiparameter particle sensing, while full polarization recovery, independently controlled material inference, and externally validated classification remain beyond the demonstrated scope.

## Figures and Tables

**Figure 1:**
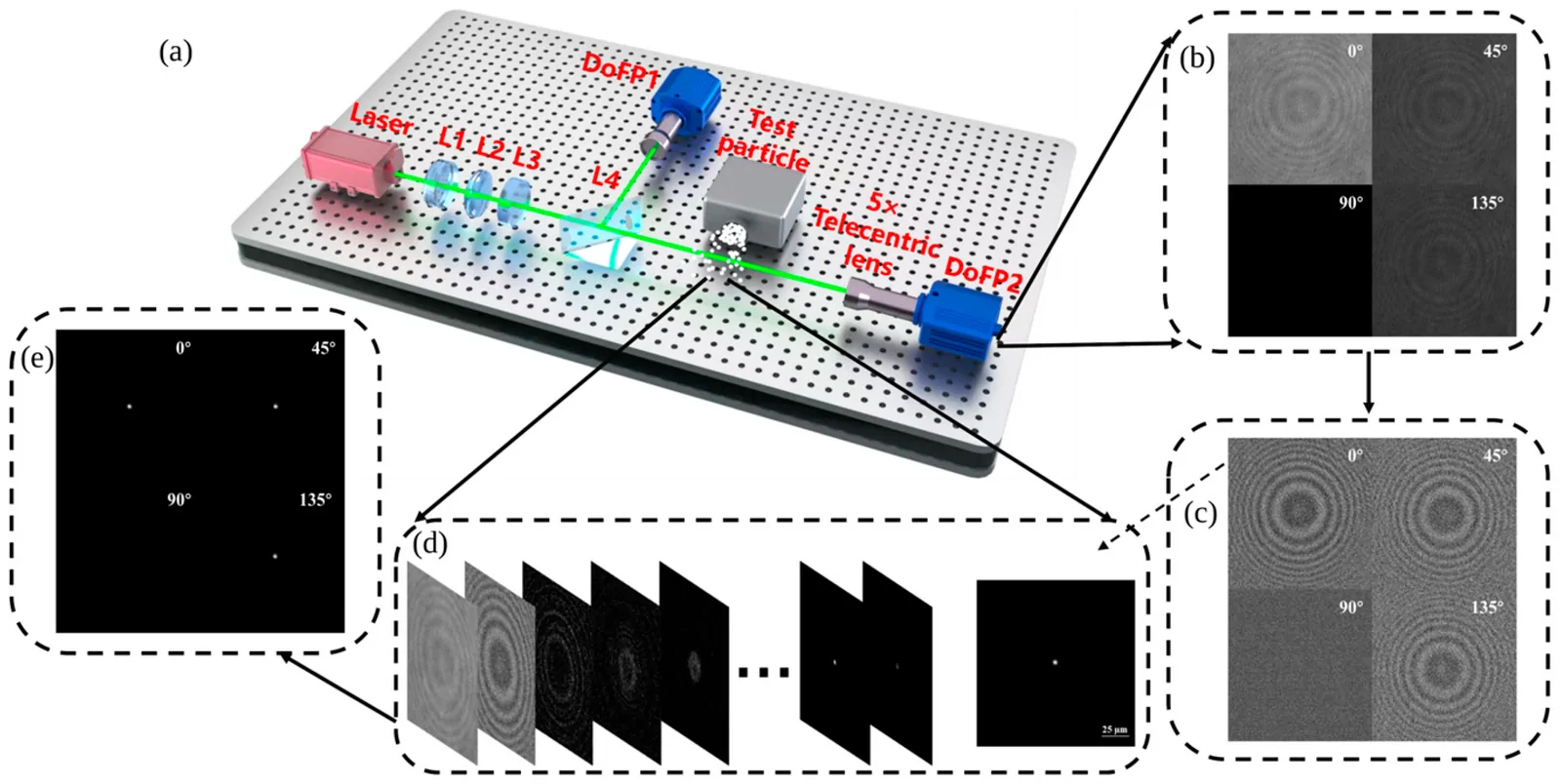
(**a**) Polarization-resolved digital holographic sensing system: Laser, 520 nm pulsed laser; L1, beam-expander lens; L2, collimating lens; L3, linear polarizer; L4, beam splitter; division-of-focal-plane (DoFP) cameras: DoFP1, incident-polarization monitoring camera; DoFP2, measurement polarization camera; 5× telecentric lens. (**b**) Polarization-resolved hologram of droplets. (**c**) Background-subtracted polarization-resolved hologram. (**d**) Numerical particle-refocusing sequence, where the ellipsis indicates omitted intermediate reconstruction planes. (**e**) Reconstructed in-focus particle image.

**Figure 2:**
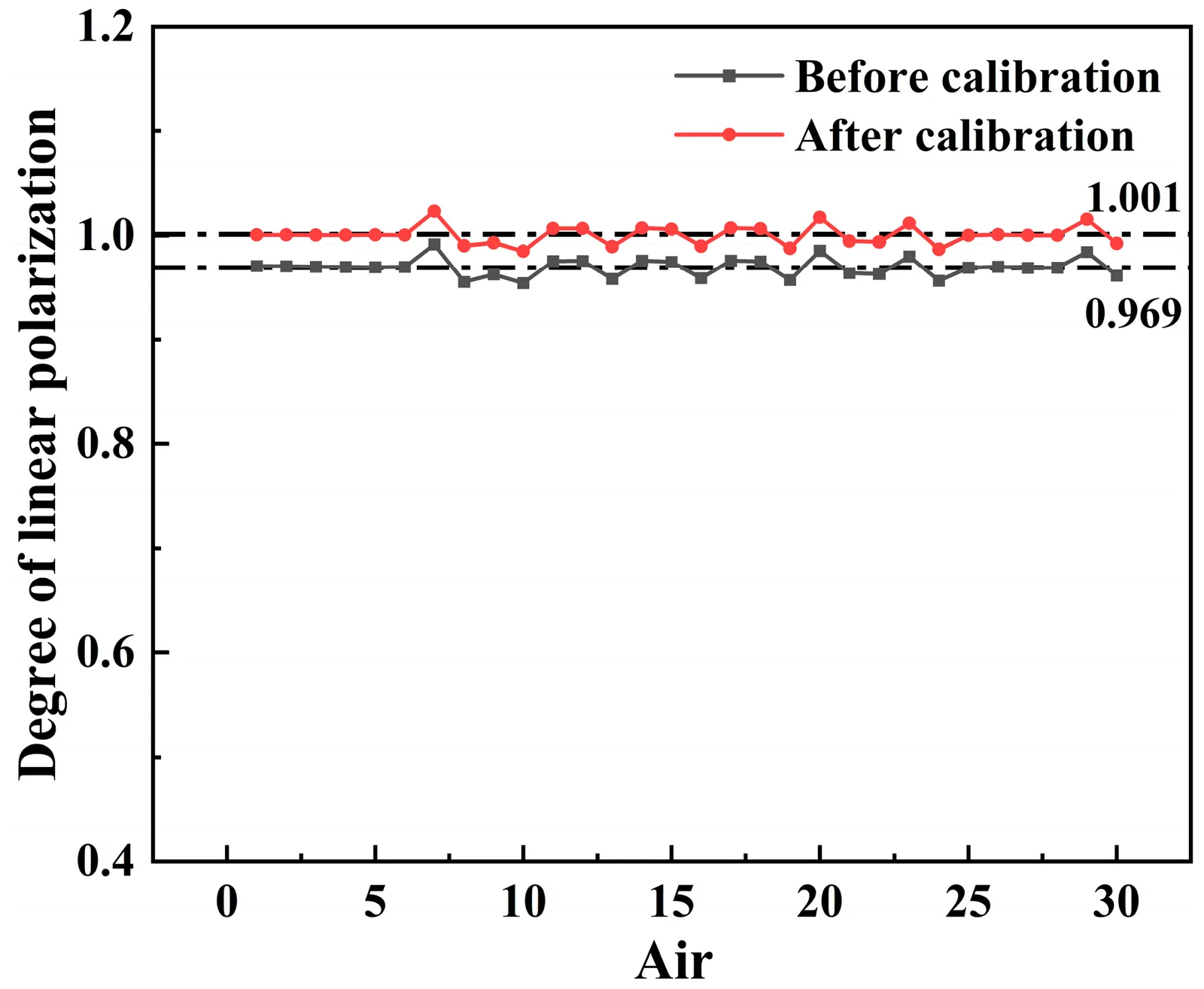
Degree of linear polarization (DoLP) measured in a particle-free region before and after calibration. The horizontal axis denotes the measurement index.

**Figure 3:**
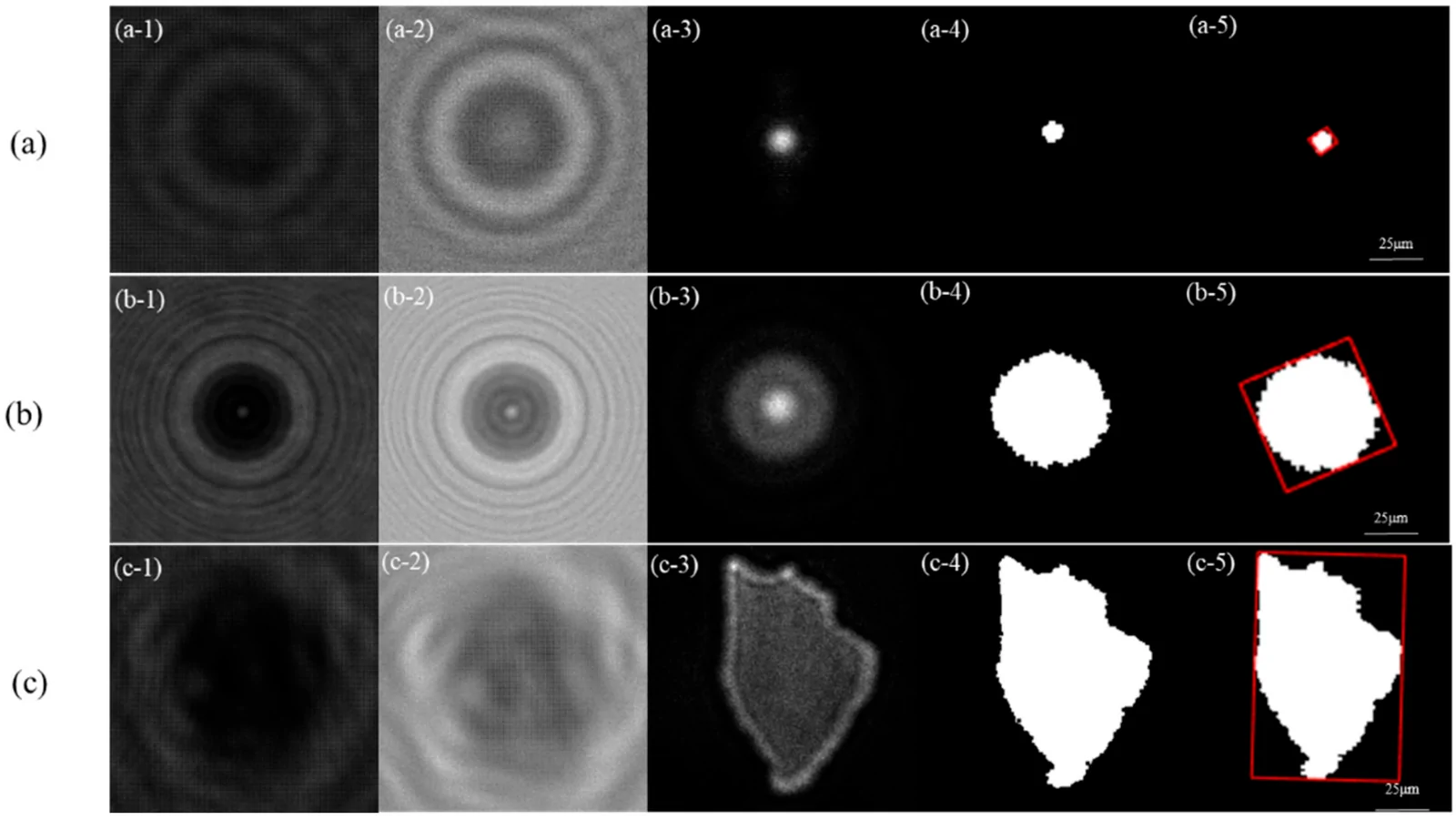
Morphological processing results for three representative particles: (**a**) a 10 μm borosilicate microsphere; (**b**) a 50 μm soda-lime glass microsphere; and (**c**) a sand grain. (**a-1**,**b-1**,**c-1**), raw particle holograms; (**a-2**,**b-2**,**c-2**), background-subtracted particle images; (**a-3**,**b-3**,**c-3**), reconstructed in-focus particle images; (**a-4**,**b-4**,**c-4**), segmented particle masks; (**a-5**,**b-5**,**c-5**), minimum-area bounding rectangles.

**Figure 4:**
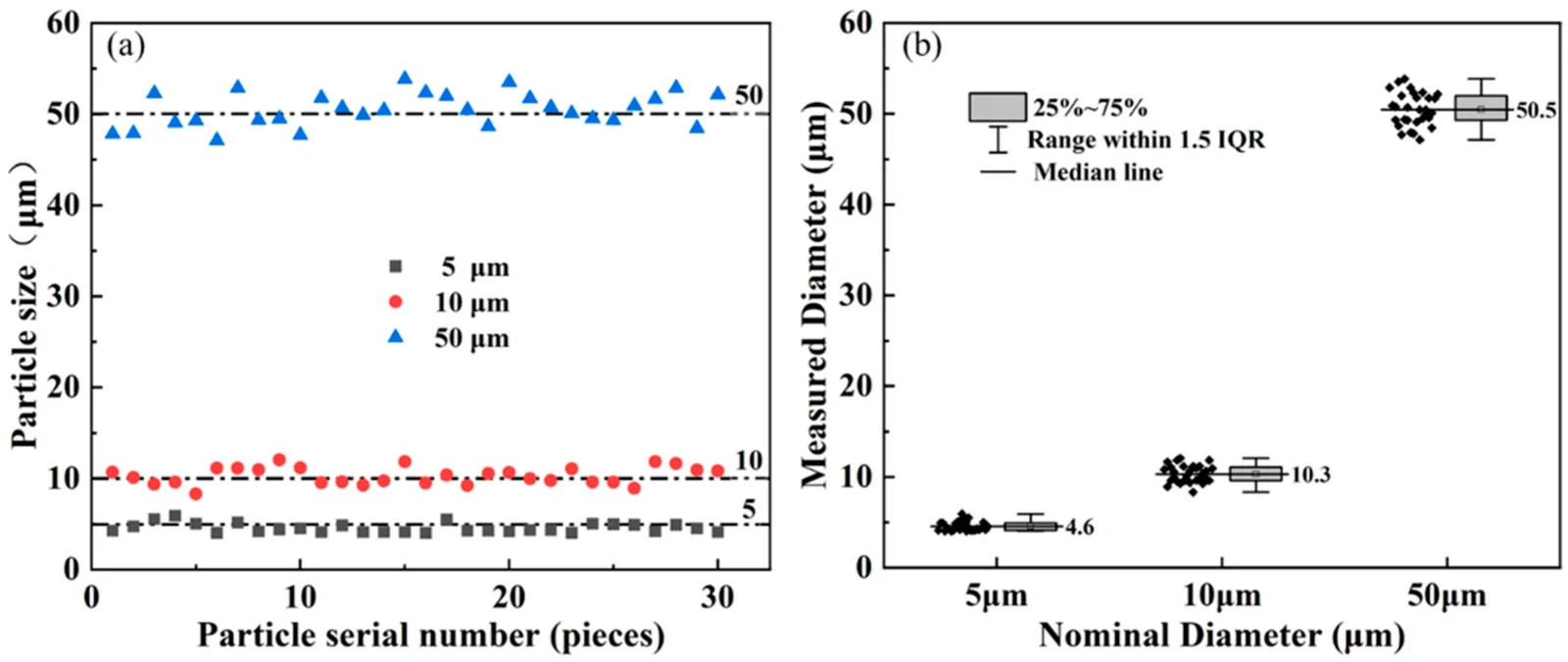
Particle-size measurements of the three standard microsphere groups: (**a**) measured equivalent diameters for nominal 5, 10, and 50 μm particles; (**b**) distribution of measured diameters for the three nominal size groups. In (**b**), boxes represent the 25th–75th percentiles, whiskers indicate the range within 1.5 × interquartile range (IQR), horizontal lines denote medians, and squares denote means.

**Figure 5:**
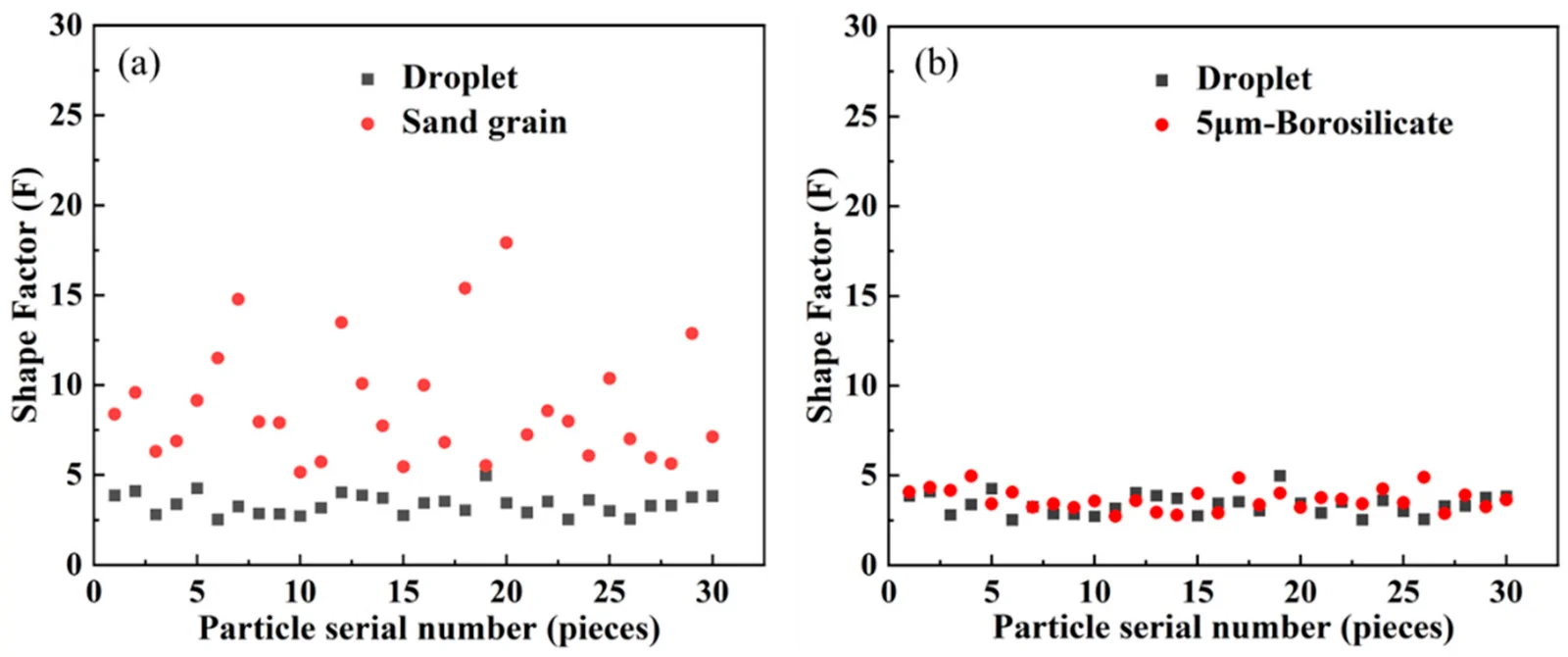
Shape-factor *F* distributions for (**a**) droplets and sand grains and (**b**) droplets and 5 μm borosilicate microspheres.

**Figure 6:**
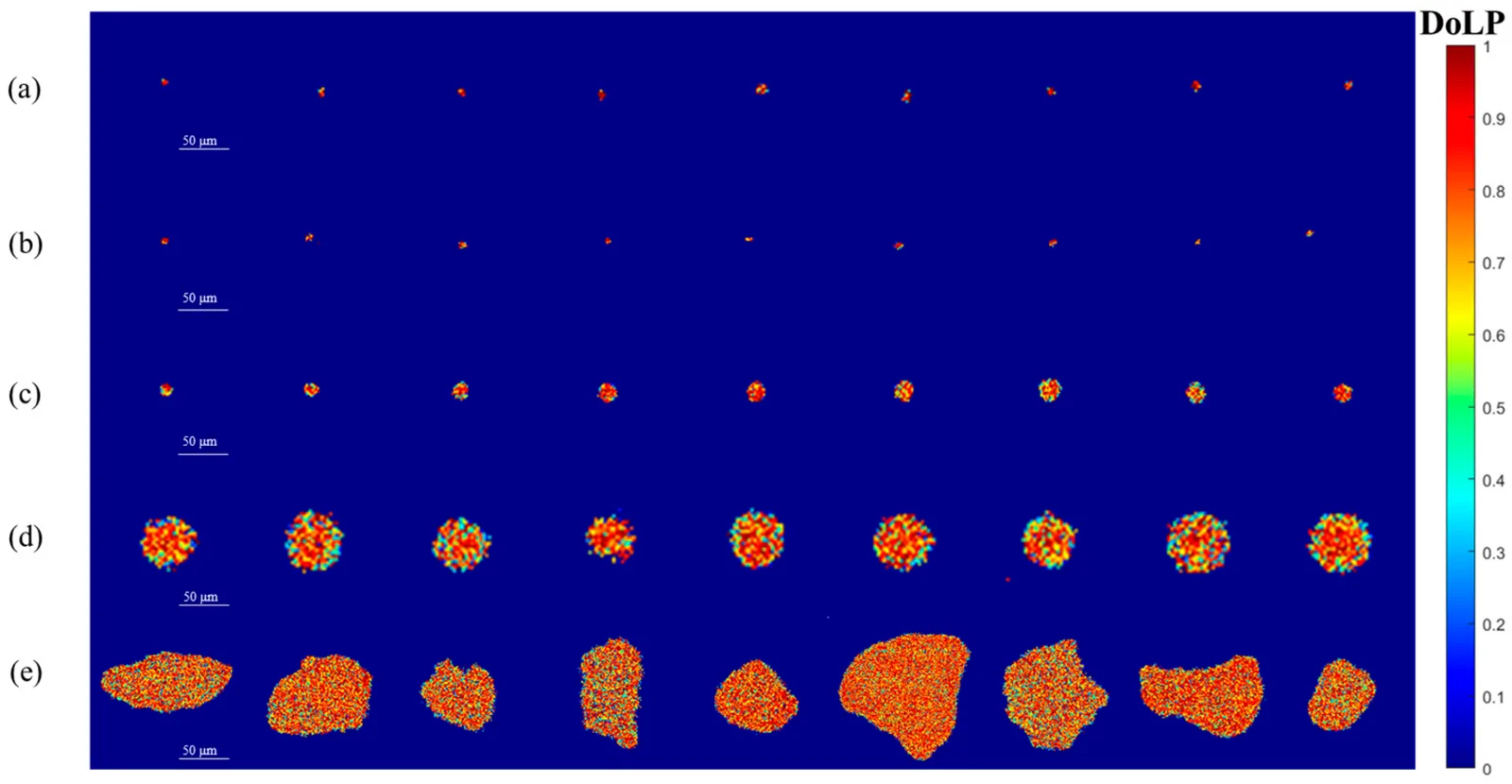
Representative DoLP maps of the investigated particle groups: (**a**) droplets (*n* = 1.33, approximately 5 μm); (**b**,**c**) borosilicate microspheres (*n* = 1.47, 5 and 10 μm); (**d**) soda-lime glass microspheres (*n* = 1.52, 50 μm); and (**e**) sand grains (*n* = 1.50). The refractive-index values are the nominal values used for comparison in this study.

**Figure 7:**
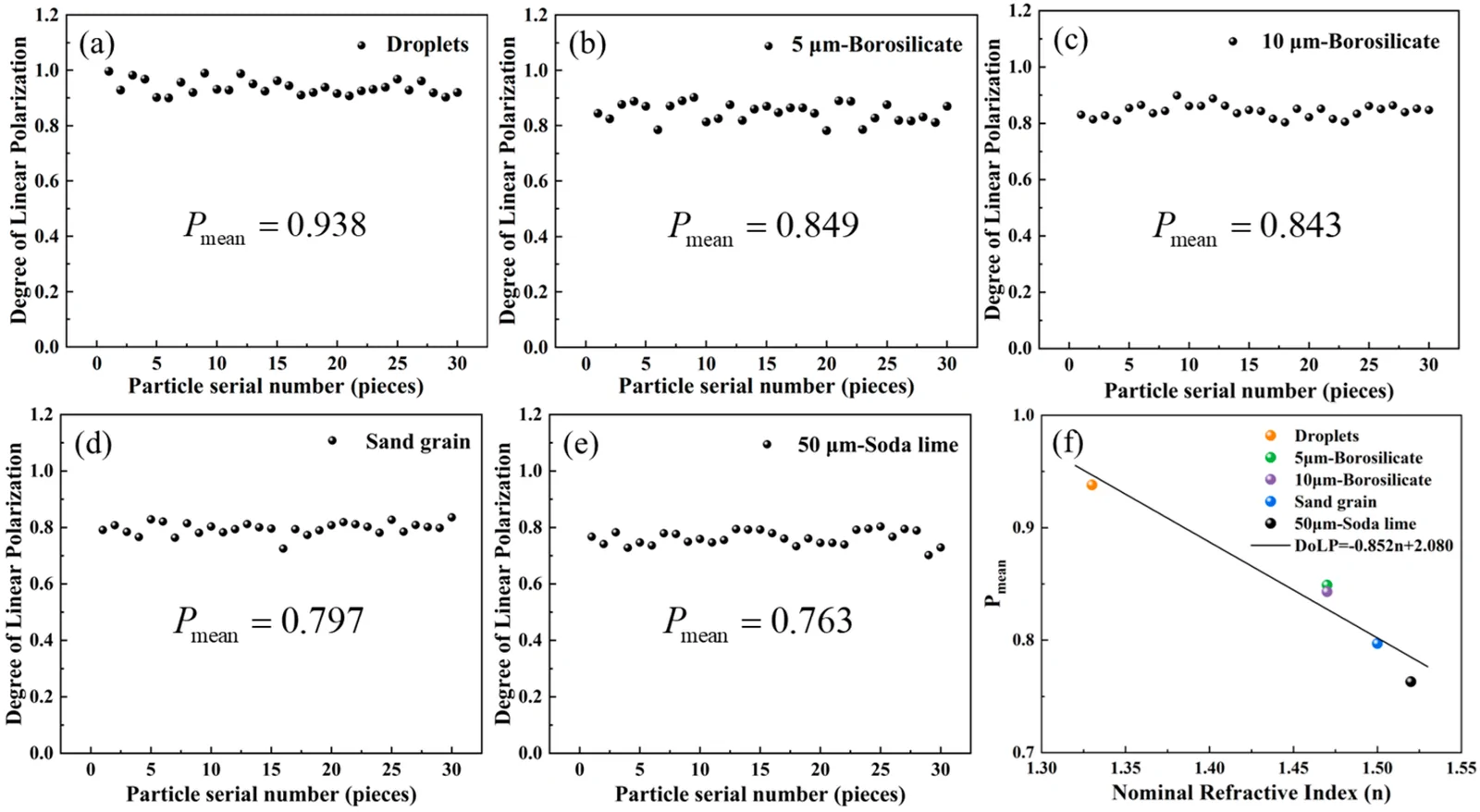
DoLP distributions for (**a**) droplets, (**b**) 5 μm borosilicate microspheres, (**c**) 10 μm borosilicate microspheres, (**d**) sand grains, and (**e**) 50 μm soda-lime microspheres; (**f**) exploratory regression of group-mean DoLP against the adopted nominal refractive indices.

**Figure 8:**
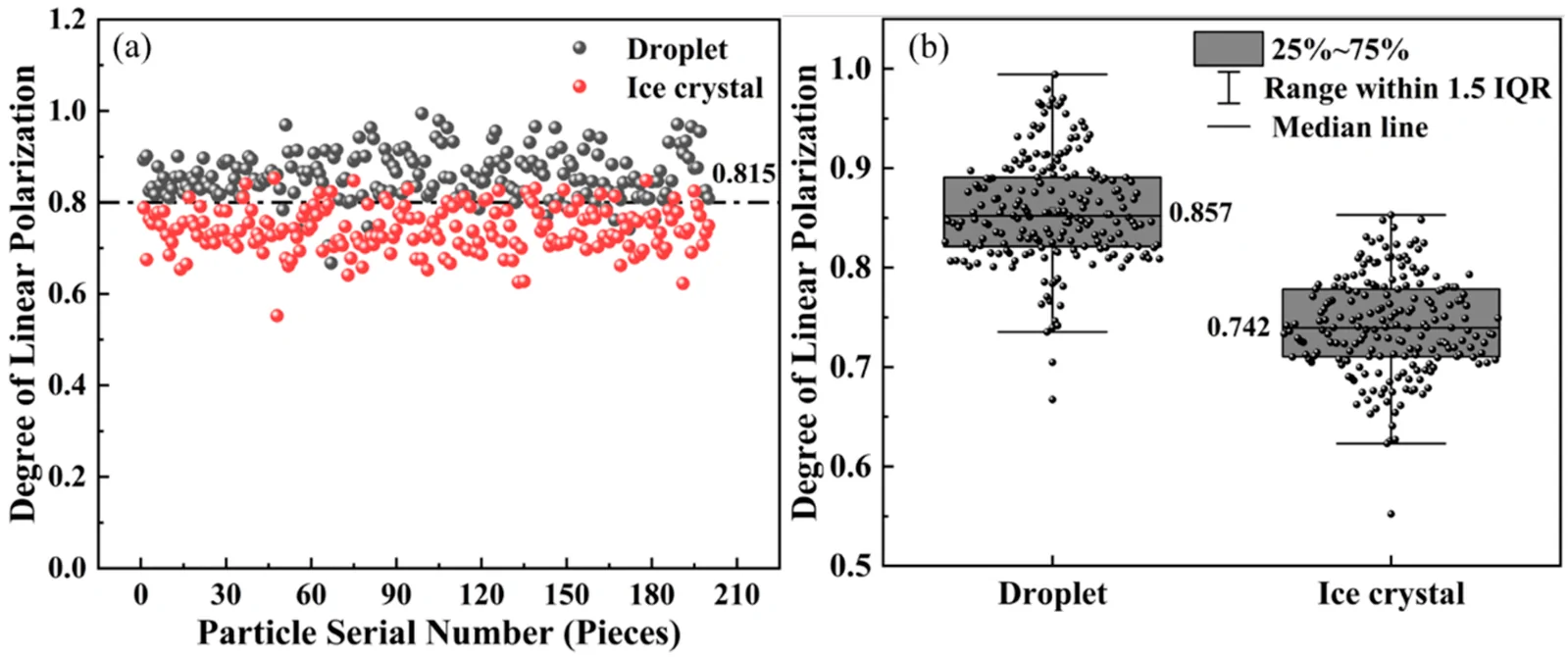
DoLP values of droplets and ice crystals measured in the ice-cloud chamber: (**a**) scatter plot of DoLP values; (**b**) box plots of the DoLP distributions. Circular markers represent individual particle DoLP measurements.

**Figure 9:**
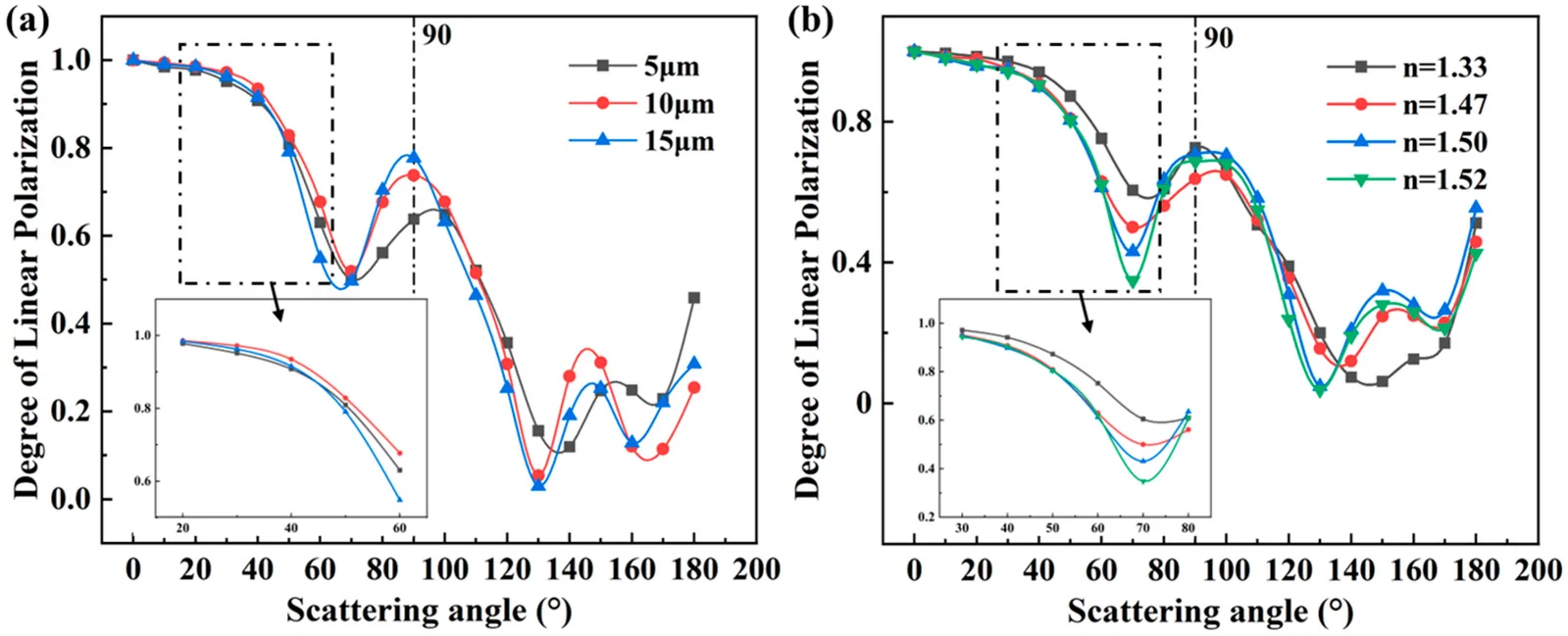
T-matrix simulations of the degree of linear polarization (DoLP) as a function of scattering angle at 520 nm: (**a**) nominal particle diameters of 5, 10, and 15 μm at a fixed refractive index of *n* = 1.47; (**b**) nominal refractive indices of 1.33, 1.47, 1.50, and 1.52 at a fixed particle diameter of 5 μm.

**Table 1:** Three-dimensional positions and projected morphological parameters of the representative particles shown in Figure 3.

	*X* (mm)	*Y* (mm)	*Z* (mm)	Diameter (μm)	Minor Axis (μm)	Major Axis (μm)	Perimeter (μm)	Area (μm^2^)	*F*
a	0.297	1.143	3.8	8.94	8.01	8.546	27.13	62.84	3.69
b	0.179	1.079	2.6	48.71	47.3	47.84	183.29	1863.5	4.71
c	1.363	1.039	5.4	90.53	77.2	118.69	369.97	6437.3	6.82

## Data Availability

The data presented in this study are available from the corresponding author upon reasonable request.

## Abbreviations

The following abbreviations are used in this manuscript:

PRDH	Polarization-Resolved Digital Holography
DIH	Digital In-Line Holography
DoFP	Division of Focal Plane
DoP	Degree of Polarization
DoLP	Degree of Linear Polarization
